# Enterobacter sp. Strain SM1_HS2B Manifests Transient Elongation and Swimming Motility in Liquid Medium

**DOI:** 10.1128/spectrum.02078-21

**Published:** 2022-06-01

**Authors:** Zhiyu Zhang, Haoming Liu, Hamid Karani, Jon Mallen, Weijie Chen, Arpan De, Sridhar Mani, Jay X. Tang

**Affiliations:** a Brown University, Physics Department, Providence, Rhode Island, USA; b Albert Einstein College of Medicine, New York, New York, USA; Forschungszentrum Jülich GmbH

**Keywords:** bacterial elongation, septa, flagella, swimming, swarming, hyperswarmer, *Enterobacter*, bacterial growth, bacterial motility, bacterial swarming, bacterial swimming, cell division, flagellar, hyperswarming

## Abstract

Many species of bacteria change their morphology and behavior under external stresses. In this study, we report transient elongation and swimming motility of a novel Enterobacter sp. strain, SM1_HS2B, in liquid broth under a standard growth condition. When growing in the Luria-Bertani medium, HS2B cells delay their cell division and elongate. Although transient over a few hours, the average cell length reaches over 10 times that of the stationary-state cells. The increase is also cumulative following repeated growth cycles stimulated by taking cells out of the exponential phase and adding them into fresh medium every 2 hours. The majority of the cells attain swimming motility during the exponential growth phase, and then they lose swimming motility over the course of several hours. Both daughter cells due to division of a long swimming cell retain the ability to swim. We confirm that the long HS2B cells swim with rigid-body rotation along their body axis. These findings based on microscopic observation following repeated cycles of growth establish HS2B as a prototype strain with sensitive dependence of size and motility on its physical and biochemical environment.

**IMPORTANCE** Bacteria undergo morphological changes in order to cope with external stresses. Among the best-known examples are cell elongation and hyperflagellation in the context of swarming motility. The subject of this report, SM1_HS2B, is a hyperswarming strain of a newly identified species of enterobacteria, noted as Enterobacter sp. SM1. The key finding that SM1_HS2B transiently elongates to extreme length in fresh liquid medium offers new insights on regulation in bacterial growth and division. SM1_HS2B also manifests transient but vigorous swimming motility during the exponential phase of growth in liquid medium. These properties establish HS2B as a prototype strain with sensitive dependence of size and motility on its physical and biochemical environment. Such a dependence may be relevant to swarming behavior with a significant environmental or physiological outcome.

## INTRODUCTION

Many species of bacteria change their morphology in certain environments (1). The most common occurrence for rodlike bacteria is that they grow longer under external stresses, including under predation (2), starvation (3), limited availability of oxygen (4), temperature change (5), DNA damage or alteration (6), and altered osmotic pressure (7). The elongation process is indicative of a delay in cell division, allowing the cells to cope with or adapt to the new environment (8, 9). During this process, the cells may pause division for hours and grow up to as much as over a hundred times their normal length (1).

Aside from external stress factors causing elongation, studies also found that many species of bacteria elongate as they manifest a collective mode of motility called swarming (9). Swarming motility requires flagella, and the elongated cells tend to be hyperflagellated. Both elongation and hyperflagellation may be beneficial for coordinated motion in high cell density. Given the environmental and physiological benefits of swarming motility, such as eﬃcient spreading over surfaces (10–12) and enhanced antibiotic resistance (13–15), the process of elongation and hyperflagellation may have developed, been fine-tuned, and become genetically regulated through the course of evolution. Therefore, morphological changes of bacterial length and its coupling to motility form an important subject of study for many species of flagellated bacteria.

Recently, two unique Enterobacter family members, the Enterobacter sp. strains SM1 and SM3, have been identified in the feces of normal and inflamed mice, respectively (16). A hyperswarming strain of SM1, named SM1_HS2B (referred to as HS2B here), has been derived through serial passage of cells from the SM1 swarm edge onto a soft agar plate (16, 17). Unlike other species of bacteria that elongate during swarming and, to the best of our knowledge, readily revert to regular size once grown in liquid media (7, 9, 18–23), HS2B is found to persist in growing elongated upon repeated regrowth in fresh LB medium before dividing into its short, steady-state form.

This study focuses on changes in cell length and swimming motility of HS2B during its growth in liquid medium. Following a particular protocol of repeated 2-h cycles of regrowth, we readily obtain cells that reach over 50 μm in average length. Most of the elongated bacteria attain swimming motility in 2 to 3 h, lasting over the course of several hours. By measuring the time course of cell elongation and swimming motility, our findings offer mechanistic insight toward understanding bacterial morphology and motility.

## RESULTS

### HS2B cells transiently elongate during fresh regrowth in liquid medium.

**FIG 1:**
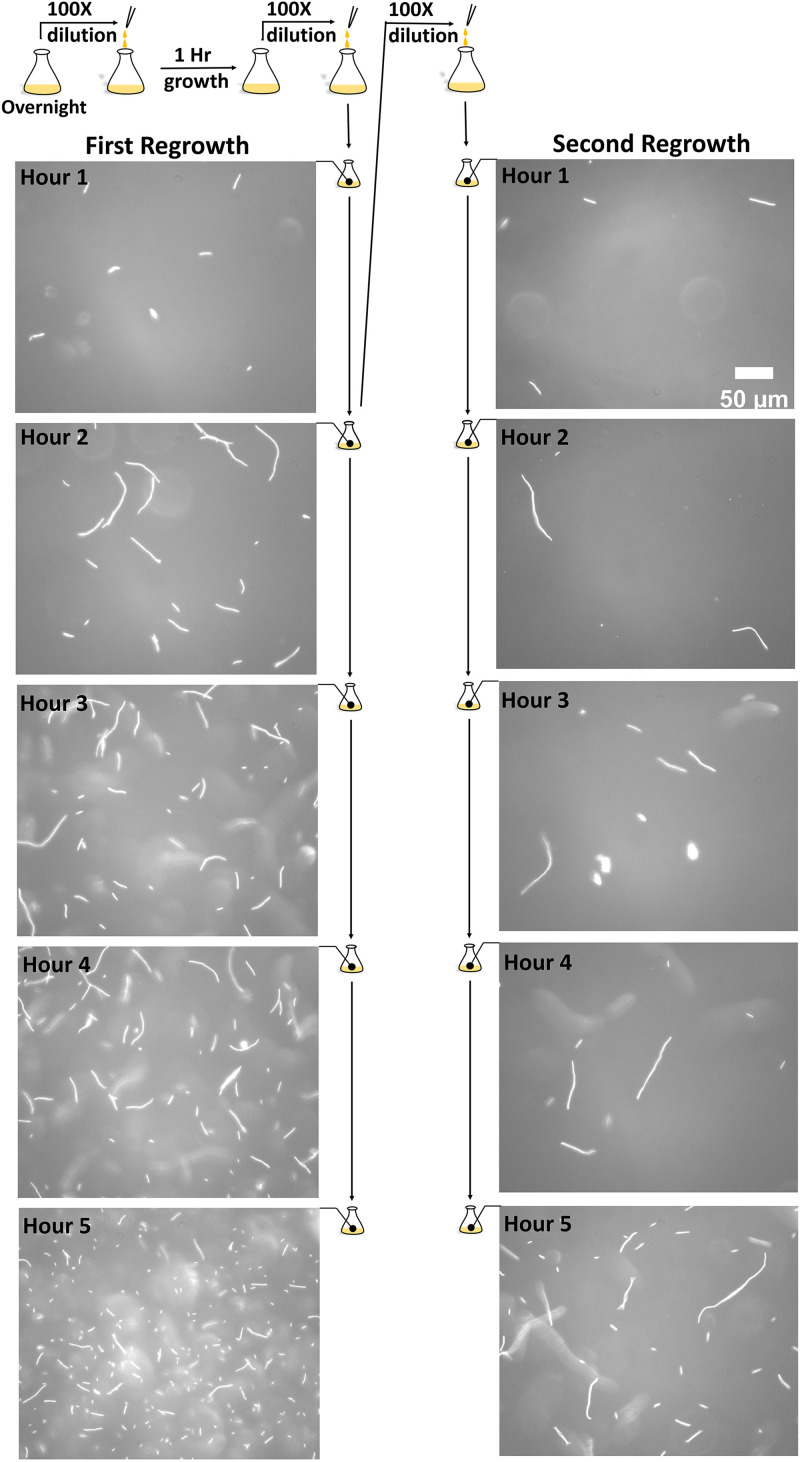
A schematic illustration of the experiment and representative images at key time points. Cartoons of flasks depict the experimental procedure. The phase-contrast microscopic images show both relative scarcity and characteristic bacterial lengths at the indicated time points of examination. The scale bar is 50 *μ*m, which applies to all 10 images.

Isolated as a hyperswarming strain of Enterobacter sp. SM1, SM1_HS2B cells (2.5 ± 1.5 *μ*m; *n* = 200) were found to be nearly twice the average length of SM1 cells (1.3 ± 0.4 *μ*m; *n* = 200), both taken from overnight liquid culture. Here, relatively large standard deviations are indicative of notable fractions of long cells, especially for HS2B. With 200 cells measured in each group, the standard errors were much smaller, so that the difference in average length between HS2B and SM1 is highly reliable. This difference makes sense since HS2B is expected to be longer because of being isolated based on its hyperswarming property. However, when we tested whether the elongation is reversible by serially regrowing HS2B cells in Luria-Bertani (LB) broth, we found surprisingly that the cells did not become shorter as expected but, instead, grew even longer. Upon repeated, 2-h cycles of regrowth from the overnight culture, the HS2B bacteria elongated in length with some exceeding 100 μm, which is approximately 40 times the length observed for most cells in the overnight growth. Over the course of several hours, however, the majority of cells decreased in length down to the level of overnight growth (Fig. 1, left column).

We also performed limited control experiments on SM1, from which HS2B was derived, and Escherichia coli. This property of extreme cell elongation does not occur under the same experimental condition for SM1 or E. coli. Instead, the average cell length increased to 3 to 4 *μ*m for SM1 during the first hour of growth and to approximately 5 μm for E. coli during the first 2 h of growth. Following onset of cell division, the average cell length then dropped down to the SM1 and E. coli stationary values of 1.3 *μ*m and 1.5 *μ*m, respectively, in several hours (data not shown). We did not see cells longer than 10 *μ*m for either SM1 or E. coli.

To examine the unique elongation behavior exhibited by the strain HS2B and rule out overpopulation or starvation effects, we designed additional experiments to test if the behavior is robust and whether it persists in repeated cycles of growth. Specifically, we diluted the culture of the first regrowth toward the end of the 2nd hour by 100-fold into fresh LB, starting what we refer to as the second regrowth. Fewer cells are shown in the images in the right column of Fig. 1, mainly due to the 100-fold dilution to start the second regrowth. Nevertheless, by moving the microscope stage to view different regions of several samples, we were able to image hundreds of cells at each time point to reliably determine the average cell length and follow its change over time.

**FIG 2:**
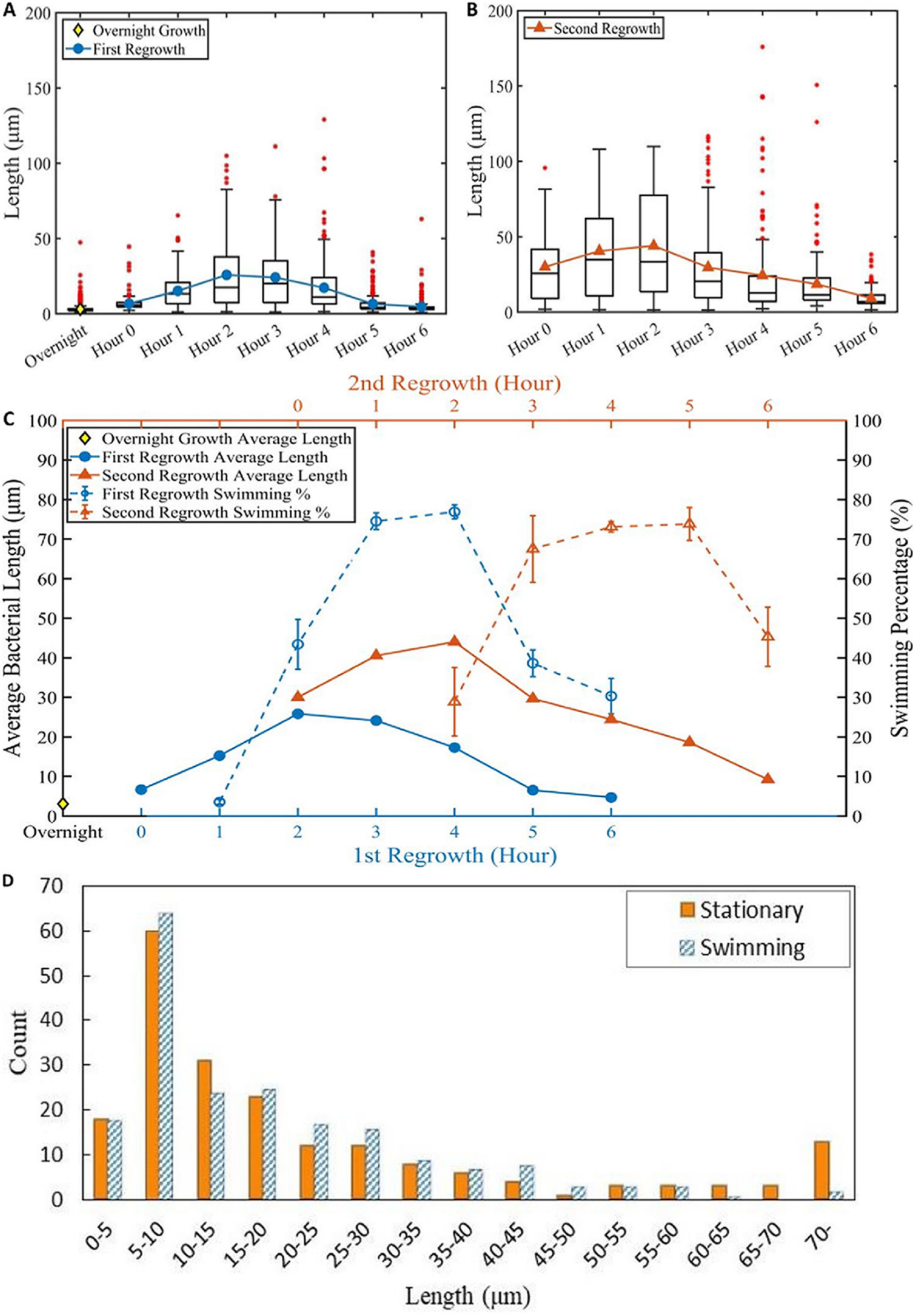
Distributions of the cell length and the swimming percentage versus time following two cycles of regrowth. (A) Box plot for the distribution of the cell length following the first cycle of regrowth. The blue circles indicate the average lengths. (B) Box plot for the distribution of the cell length following the second regrowth. Note that Hour 0 of the second regrowth is measured immediately after dilution of Hour 2 of the first regrowth. The orange triangles indicate the average length. For both box plots, the red asterisks are outliers; the horizontal black line inside the box represents the median. Over 100 cells were measured at each time point. (C) Comparison of average length and swimming percentage versus time between the first and the second regrowth. The average length peaked about 2 h following each growth; the swimming percentage peaked about 2 h afterward. The error bars show standard deviations in percentages determined over 3 separate experiments, counting swimming and stationary cells at each hour. The total counts at each hour from the 3 experiments are listed in Table 1. (D) Histograms for the number of stationary and swimming cells 3 h following the first regrowth. The distributions are similar with the exception of a few extremely long cells mostly being stationary.

The average length of HS2B, measured hourly following both cycles of regrowth, increases over time, with the peak occurring around 2 h after dilution for both the first regrowth and the second regrowth (Fig. 2A and B). The median length is smaller than the average, which is skewed by a subset of cells that have grown to extreme lengths. After 2 h, both cycles of regrowth show decay in average length, while the longest cells (129.0 *μ*m for the first regrowth and 175.9 *μ*m for the second) were found at the 4th hour for both cycles of regrowth (Fig. 2A and B). This result suggests that although the majority of cells start to divide about 2 h following each regrowth, a few outliers continue to elongate and delay their cell division until after around 4 h. After 5 or 6 h of the first regrowth, however, the average cell length appears to be reduced to a range comparable to overnight growth. Similar to the first regrowth, the average cell length increases within the first 2 h of the second regrowth and then drops in the following 4 or 5 h, approaching the short cell length as most cells exist in their stationary state (see the orange triangles in Fig. 2B).

The average length values of both cycles of regrowth are replotted in close comparison in Fig. 2C, showing that the average cell length in the second regrowth is greater than in the first regrowth at all subsequent hours. These data suggest delay of cell division by about 2 h, following each regrowth. The delay appears cumulative, giving even longer average cell length 2 h following dilution for the second regrowth than for the first regrowth. The average length following repeated regrowth reaches about 55 *μ*m following a third regrowth, but then it starts falling following the fourth and fifth regrowth (see Fig. S1 in the supplemental material), suggesting that replenishing fresh medium every 2 h does not prevent the cell division indefinitely. Nevertheless, the trait of transient elongation is robust in the sense that it manifests the same behavior upon fresh growth and regrowth from the overnight culture, including that frozen and stored at −80°C and thawed for new growth.

### HS2B cells attain swimming motility during fresh regrowth in liquid medium.

Although late-log or overnight growth HS2B cells are nonmotile, most if not all cells develop an ability to swim in fresh LB during the phase of exponential growth, and the swimming motility lasts a few hours until the late log phase. This behavior applies to both short and long HS2B cells. Whereas short HS2B cells frequently pause and change their swimming directions, elongated cells move vigorously and, with rare exceptions, unidirectionally (Movie S1). By taking short movies, we were able to distinguish swimming cells from nonswimming cells and determine the percentage of cells swimming at each hour (Fig. 2C). Similar to the average cell length, the swimming percentage also shows a transient increase following each regrowth, reaching peak values of over 70% 3 or 4 h after initiation of each regrowth (Table 1). Comparing the average cell length and swimming percentage plots for each cycle, there is approximately a 2-h delay in reaching the peak of swimming percentage following the peak of the average length.

**TABLE 1:** Hourly counts of stationary and swimming cells^*a*^

Hour	First regrowth			Second regrowth		
	No. of cells		% swimming	No. of cells		% swimming
	Stationary	Swimming		Stationary	Swimming	
1	242	9	3.6	53	36	40.5
2	303	232	43.4	133	54	28.9
3	298	865	74.4	151	315	67.6
4	304	983	76.4	260	719	73.4
5	884	556	38.6	326	914	73.7
6	1,555	675	30.2	458	359	43.9

a The numbers counted and the percentages of swimming are listed from hour 1 to hour 6 following the first and second regrowth, respectively.

To examine if cells of different lengths develop different propensities to swim, we counted 200 swimming cells versus 200 nonswimming cells and broke them down in narrow ranges of 5 *μ*m in length. We did the comparison at the selected time point of 3 h after the first regrowth as both the average cell length and the swimming percentage were near their peak values. The results show very similar histograms (Fig. 2D), with the exceptions of a few extremely long cells (longer than 70 *μ*m). These exceptions indicate a delayed start of swimming of extremely long cells, which we observed under the microscope at multiple time points. The main conclusion here is that cells of various lengths attain swimming motility over time but, at a particular time point, there is no strong correlation between the cell length and its propensity to swim.

### Elongated cells manifest delayed onset in swimming.

**FIG 3:**
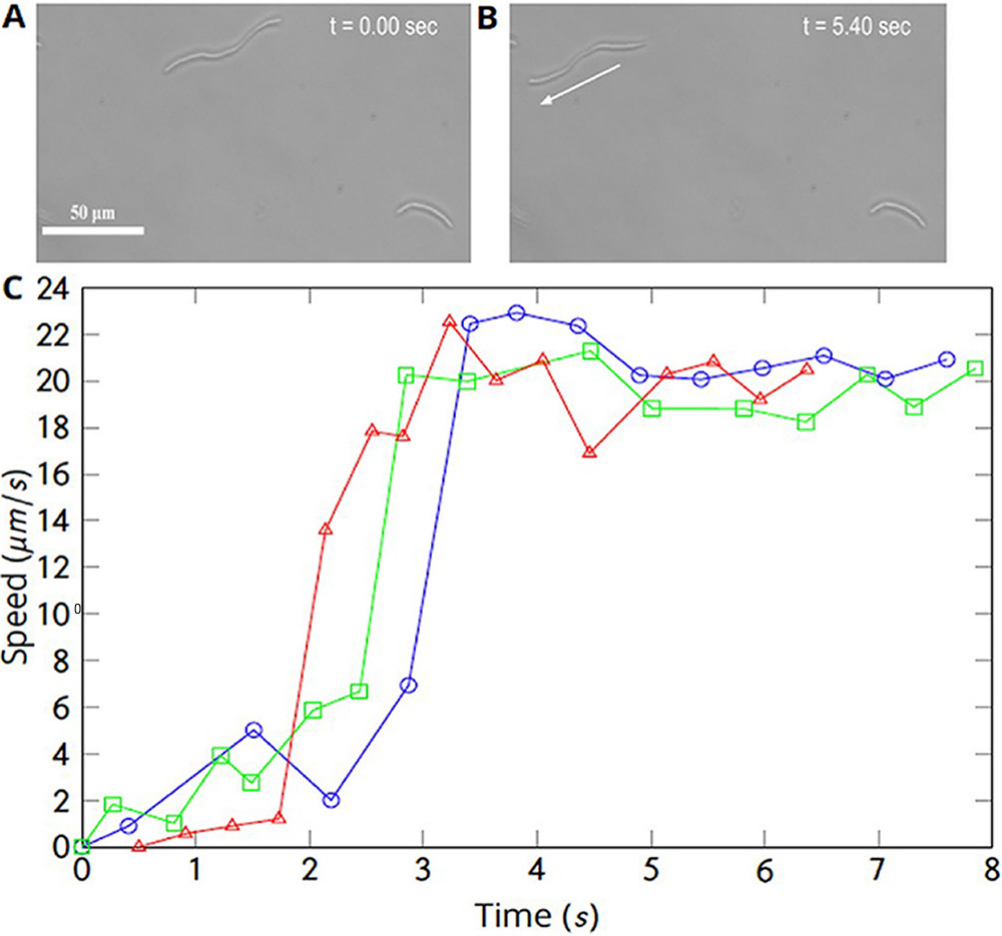
Live captures of speed changes of long cells from stationary to swimming. (A and B) Microscopy images taken at 0.00 and 5.4 s in a short movie clip. (C) Changes of speed for three cells from stationary to swimming. The speed approaches a nearly constant steady-state value of roughly 20 *μ*m/s in about 2 s after each of these cells started to swim. The blue symbols in panel C are measurements for the cell noted in panels A and B. The red and green symbols represent two other cells.

In order to understand why the percentage of swimming cells peaked after the average cell length, we observed each sample under the optical microscope over several minutes, noting two interesting behaviors. First, many long cells displayed uncoordinated motion prior to swimming. Then, after some time of jiggling or body rotation without much translation, they suddenly started swimming, i.e., moving in significant speed along their bod*y* axes. One example cell is shown by Movie S2, with images at two time points shown in Fig. 3. Tracking cells from stationary state to swimming, the speeds of three sample cells (61.1 *μ*m in blue, 43.9 *μ*m in green, and 59.0 *μ*m in red) are plotted (Fig. 3C). Data from all three cells show a sudden onset of swimming behavior, and in each case the swimming speed approaches a steady-state value within 2 s after the onset of swimming motion.

**FIG 4:**
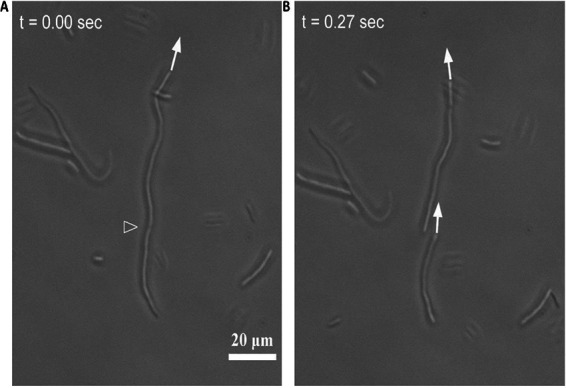
Microscopy images showing a swimming, elongated cell divided into two swimming cells. The filled arrow points to the moving direction of a long cell at *t* = 0, which points at 75.3° above the horizontal axis in Cartesian coordinates. The cell speed was 46 *μ*m/s. The hollowed arrowhead points at the location of division. The right image was taken 0.27 s later and shows the two daughter cells right after the division. The top cell is moving at 49.0-*μ*m/s speed (98° from the *x* axis); the bottom cell is at 45-*μ*m/s speed (86° from the *x* axis). These speeds and directions are roughly the same as those of the cell immediately prior to division.

The second behavior we observed is that a swimming elongated cell tends to divide into two swimming daughter cells, as exemplified in Fig. 4. This dividing cell moved at 75.3° from the *x* axis in a Cartesian coordinate with a speed of 46 *μ*m/s. At the time point of 0.27 s after the first image, the elongated cell divided into two daughter cells, which moved at 97.6° with 49-*μ*m/s speed and at 86.4° with 45-*μ*m/s speed, respectively. Although the changes in speed and travel direction are small, the division of one elongated cell into two here is clearly discernible (Movie S3). We also performed an experiment to characterize the sites of division by fluorescence labeling, with images and results described in the supplemental material.

### Elongated cells swim by rigid-body rotation.

**FIG 5:**
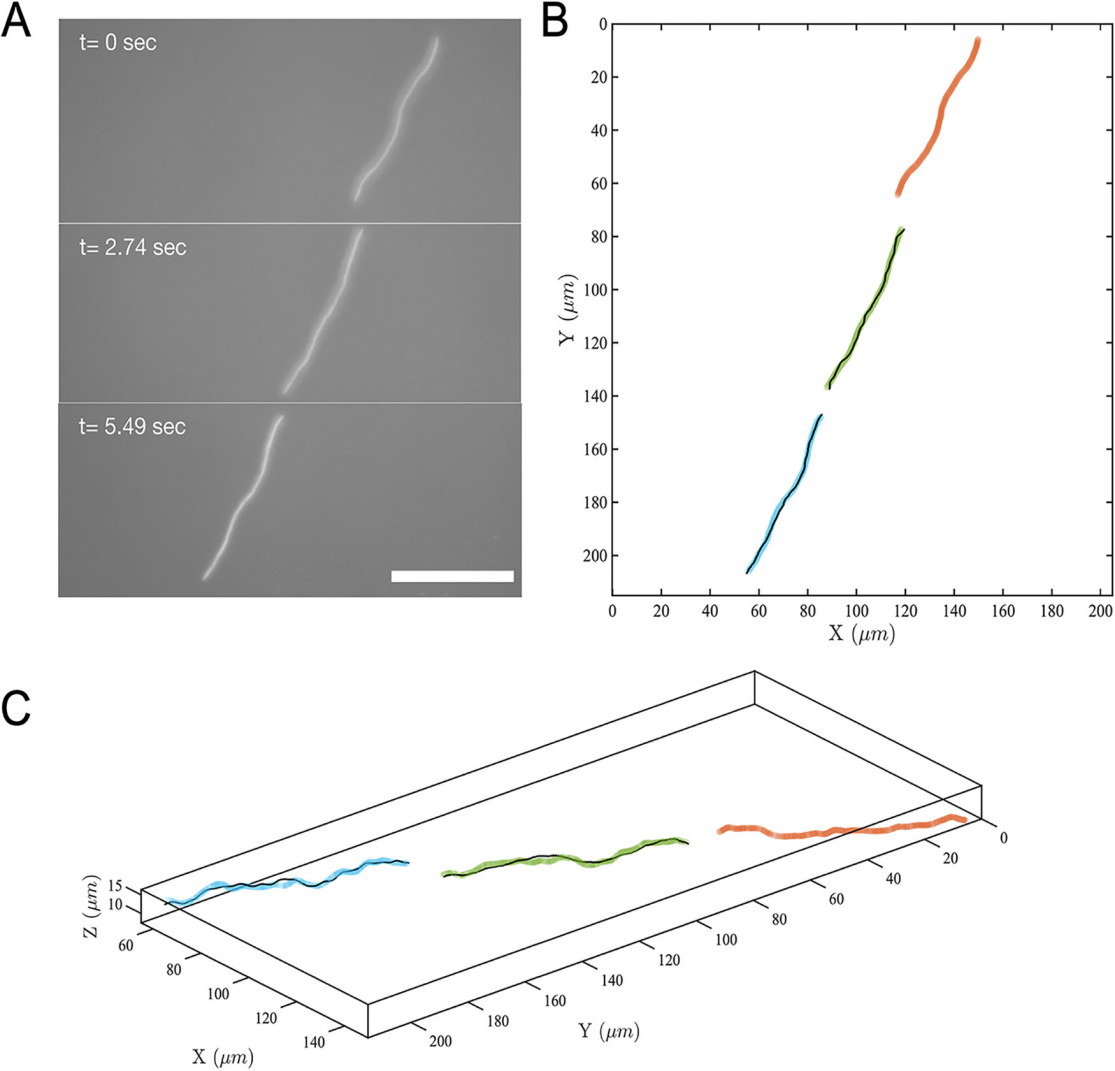
Images of an elongated cell at 3 time points with a fit of its initial 3-D shape rendered with proper rotations to show that the cell swims by rigid-body rotation. (A) Phase-contrast microscopy images of a swimming cell at three different times. Scale bar is 50 *μ*m. (B) Red, green, and blue lines, swimming cell outline in the X-Y plane at *t* = 0, 2.74, and 5.49 s, respectively; black lines, linear transformation (translation and rigid-body rotation) of cell outline at time zero (red) to times 2.74 s (green) and 5.49 s (blue). (C) Reconstructed 3-D swimming cell outline from pixel intensity at times 0 s, 2.74 s, and 5.49 s. The color scheme is the same as in panel B.

Observed under the microscope, the elongated cells manifest an intriguing form of swimming behavior. The ends of long cells that exceed tens of micrometers in length constantly undergo lateral motion, as viewed under the microscope, giving a sense of snakelike undulation (Movie S1). Since most HS2B bacteria exhibit irregularly curved shapes that are distinct from straight rods and the curvature along their contour appears to constantly change (Movie S4), we wondered in the initial stage of observation whether cells swim by lateral undulation or rigid-body rotation. We hypothesize that like most rod-shaped, flagellated bacteria, the elongated HS2B cells swim by self-rotation along their long axes rather than by lateral undulation. The body rotation is likely propelled by numerous flagella along the cell body, as shown on an electron microscopy image (Fig. S4). To test our hypothesis, we conducted cell tracking of phase-contrast microscopy images and captured both transverse (perpendicular to its long axis) and longitudinal (along its long axis) motions of the cell at different times. Toward this end, we first identified each cell’s centerline. Then, we extracted pixel intensity variation along the cell, which is linearly correlated with the depth of object away from the focal plane (Materials and Methods). The in-plane motion and reconstructed three-dimensional (3-D) motion of a swimming cell at three representative times are shown in Fig. 5 (colored lines). As cell outlines in Fig. 5A depict, selected frames are from different swimming instances and do not correspond to complete periods (or cycles) with respect to prior frames. Thus, the 2-D projection of the cell contour appears different within the different frames shown.

In order to verify that the cell motion is indeed via rigid-body rotation, we linearly transformed the initial (*t* = 0 s) cell body in 3-D to the later times (Fig. 5C, green lines) (24) (described in Materials and Methods, with additional details in the supplemental material). We found that, after linear transformation (rotation accompanied by translation), the initial cell outline matches with that at later times. This analysis confirms that elongated HS2B cells swim by rigid-body rotation.

### Swimming speed and angular speed vary as functions of cell length.

**FIG 6:**
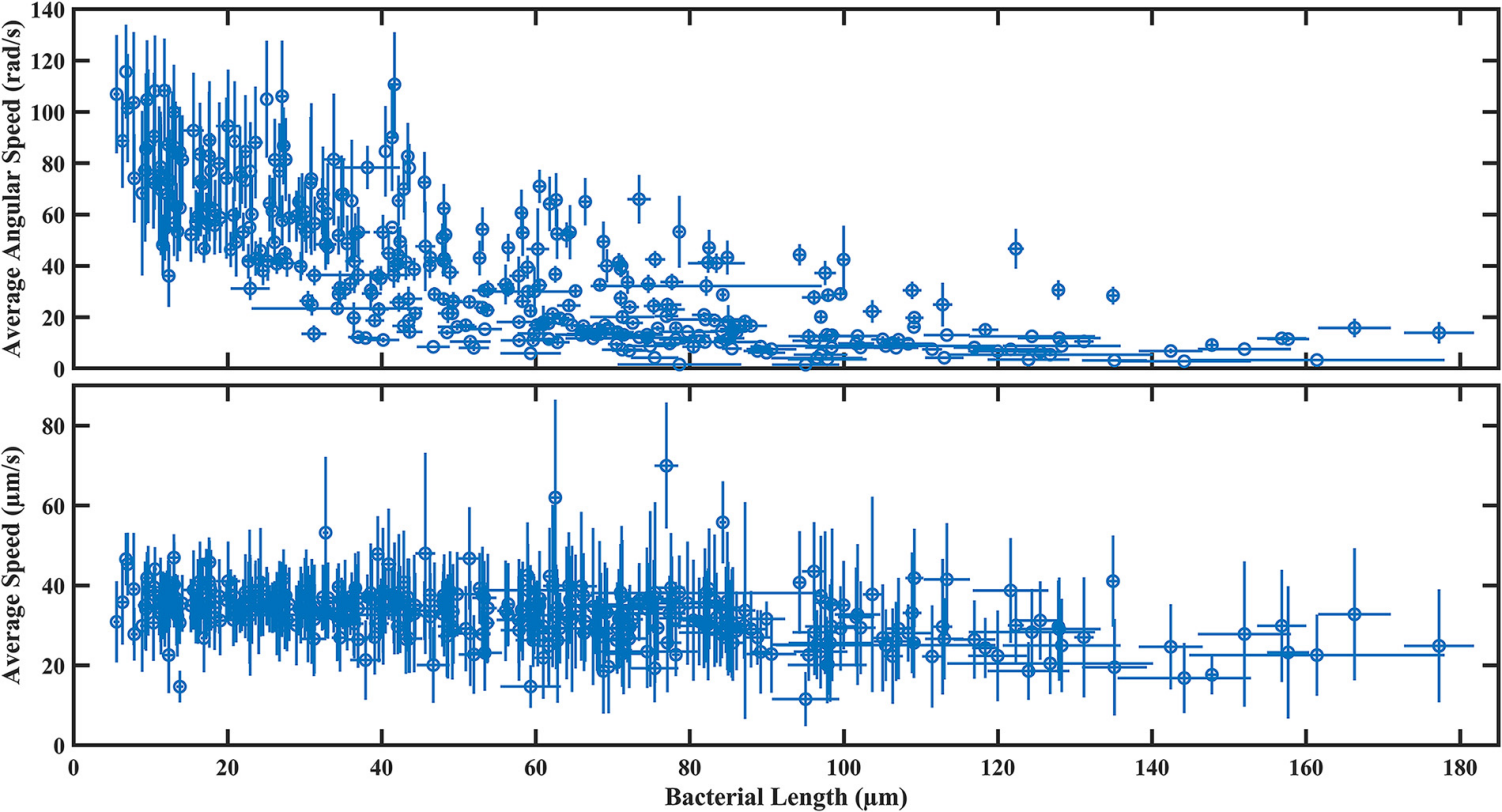
Plots for body rotation speed and swimming speed versus cell length. (Top plot) Average rotation speed of cell body with respect to its long axis. A total of 359 cells were measured. (Bottom plot) Average swimming speed of the same set of cells.

We tracked 359 swimming cells with lengths ranged from a few micrometers to over 100 *μ*m and measured how fast they swim and rotate. As the length increases, the HS2B cells’ average angular speed decreases rapidly while average linear speed remains constant at ~30 *μ*m/s up to a cell length of about 100 *μ*m (Fig. 6). The constancy of linear speed is similar to the previous work on filamentous E. coli, although the length of the cells measured then reached only ~20 *μ*m (25). For HS2B cells longer than 100 *μ*m, the speed dropped somewhat, to about 25 *μ*m/s. This moderate drop is attributable to bent shapes of extremely elongated cells, which were noted to be common but not systematically analyzed.

## DISCUSSION

Elongation due to external stress applied on different species of bacteria has been extensively studied in the past (1, 3, 8, 26). Our work here presents findings of elongation of a novel swimming- and swarming-capable HS2B strain, which is derived from a species recently identified and named Enterobacter sp. SM1 (16). It has been shown previously that Campylobacter jejuni, a microaerobic Gram-negative pathogenic bacterium that leads to human gastrointestinal diseases, elongates during its growth in liquid medium. For Campylobacter, it has been suggested that external stresses such as limited access of oxygen, change in temperature, and free radicals could be present during its growth, causing bacterial elongation (8, 27). For HS2B, however, we did not intentionally apply an environmental stress during its growth in liquid medium. Yet, we found HS2B to elongate progressively upon cycles of regrowth in fresh LB medium. The elongated HS2B cells also acquire motility during the exponential phase of growth. These properties we observed of bacterial elongation without external stress and its transient swimming motility merit further investigation.

Rod-shaped bacteria sometimes attain extreme length under conditions of environmental stress. The elongation typically occurs due to delay in dividing into daughter cells. The division requires septa, which are protein complexes responsible for separation into daughter cells (8, 28, 29). Cells that elongate without showing regular septa are referred as “filaments,” while the ones with regular septa visualized are termed “chains” (29). This definition is phenomenological, and the distinction drawn might be obscured by the quality of images acquired. Elongation to several times the regular cell length indicates a delay in division. Whether or not regular septa are observed in the elongated cell may depend on what stage of division the cell has progressed to, and often whether a particular labeling technique is sensitive enough to show the expected septa. When the division has progressed far enough, constrictions may appear at multiple locations, and by that stage, the elongated cells may be referred to as chains. The elongated HS2B cells appear to manifest a logical progression of delayed division, as the images in the supplemental material show (see Fig. S3 and S4). The fluorescence images show septa at multiple sites of elongated HS2B cells, resulting in division into short cells that are predominant in the liquid medium by the late log stage.

As HS2B increases in length, the cell body also undergoes visible changes in shape. Many cells longer than ~30 *μ*m appear to be curved (images in Fig. 1). However, for approximate calculation of the torque experienced by HS2B cell bodies of a range of different lengths, the analytic equations we used neglect the curvature effect by treating the cell, to the first order, as a long rod-like object or a prolate spheroid (see section S5 of the supplemental material text). In addition, as the curvature increases, the cell body tends to rotate a lot more slowly with respect to its long axis (see Fig. S6 and Movie S1). Nevertheless, the steep drop in rotation speed as the cell length increases does not affect the comparison for torques of the relatively short HS2B cells (~10 *μ*m) with other species of bacteria of similar length reported in the literature (Fig. S6, gray and black symbols). This comparison shows that HS2B cells in general exert somewhat larger torques as they swim than do several other bacterial species. The torque magnitude likely correlates with the number of flagella on the cell surface. In order to understand the origin of the large torque experienced by HS2B cells, one future effort could be directed at visualizing and counting the number of flagella on motile HS2B cells, as well as measuring the motor torque for this new species of bacterium.

**FIG 7:**
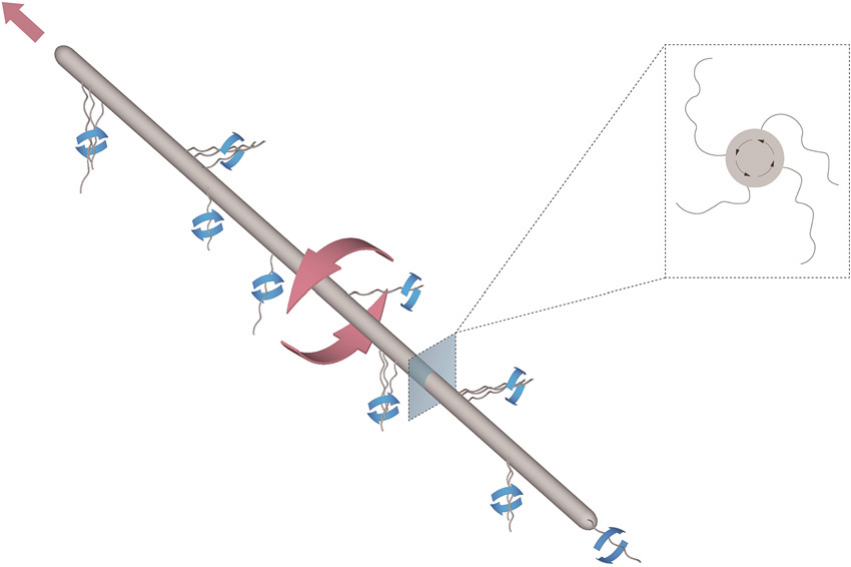
An illustrative drawing of an elongated bacterium translating and rotating in liquid medium. The overall locomotion is coupled with cell rotation along its body axis, driven by coordinated rotation of multiple flagella or flagellar bundles along the cell length. Red arrows depict rotational direction of the cell body; blue arrows show rotation directions of flagellar bundles. Inset is a cross-sectional view of the cell, depicting a spiral form necessary to cause cell body rotation.

The swimming motility of an extremely long HS2B cell is likely propelled by multiple flagella or flagellar bundles along the elongated cell body. In retrospect, given the rigid body of the elongated bacteria, it is highly unlikely for them to attain locomotion by undulation of the cell shape. Most of the flagella or flagellar bundles may align with a rearward flow as the cell moves forward (or vice versa), as illustrated in Fig. 7. Such a sliding/swimming motion requires a spontaneous alignment of multiple flagellar bundles along a long cell. The time course to meet this requirement may account for the time spent on the jiggling motion or body rotation without directional translation, i.e., swimming (Movie S2). The decreasing rotational speed versus filament length suggests that multiple flagella or flagellar bundles along the side of the long cell are not synchronized as the resultant torque rotates the elongated cell body with respect to its long axis. Alternatively, a curved shape of an extremely long cell causes a much larger viscous drag against rotation with respect to its bod*y* axis, which may also account for the steep decrease of angular rotational speed with length.

It is well known that, when inoculated on an agar surface, many species of swarming bacteria tend to elongate as they grow and spread to the edge of the swarm (9). However, to the best of our knowledge, most swarming bacterial species do not persist with the trait of becoming more elongated when they are regrown in liquid medium, unless subjected to other stress factors. The Enterobacter sp. SM1_HS2B was obtained following the same method as introduced in a study of Pseudomonas aeruginosa by van Ditmarsch et al. (30). HS2B is considered a “hyperswarming” strain as it exhibits enhanced swarming ability compared to SM1, from which it is derived (16). It is surprising that this hyperswarming strain has retained the ability to elongate even in liquid broth medium. It does so upon dilution into fresh LB, which is perhaps the simplest means of stimulation. From the point of view of swimming motility, the elongation process does not offer an obvious advantage, since the swimming speed does not increase as cell length becomes longer (Fig. 6, bottom panel). Our finding is in accordance with swarming P. aeruginosa (31), in the sense that hyperswarming cells do not have a swimming advantage in a liquid environment. Hence, our findings support the notion that elongation by inhibition of division might augment swarming motility by enhancing the cell-cell alignment due to increased cell length. The enhanced alignment among neighboring cells might also enhance their collective motion. However, further study is needed to elucidate the relationship between HS2B’s hyperswarming ability and its transient elongation and swimming motility when grown repeatedly in liquid medium.

One notable shortcoming of this report is that we have not yet annotated the genome of SM1_HS2B. The set goal of this project was biophysical behavior of transient elongation and swimming motility of this particular mutant. We do recognize, however, the need to determine the genome of this mutant, as has been done for Enterobacter sp. SM3 and SM1 in a preceding publication (16). SM1 is in fact the parent strain from which HS2B was derived by serial plating and inoculation. Although HS2B is laboratory generated, its propensity to elongation and its robust motility in liquid medium, although transient over only a few hours, indicate that it is a versatile strain. Once annotated genetically, HS2B may become a prototype strain for further studies on bacterial growth and division, as well as swimming and swarming motility.

This study also did not explore genetic regulation of the swimming motility, which peaks hours into the exponential phase of growth. Following addition of nonmotile, stationary-state bacteria into fresh medium, it takes time for them to upregulate genes and protein expressions required for growth. While elongating, the growing bacteria also need to produce enough flagella in order to attain swimming motility. The level of flagellins, the protein units of flagella, may take several hours to reach its peak, as has been shown in a previous study on E. coli (32). Similar experiments on the coupling between the expression level of flagellin and the swimming motility of HS2B over time of fresh growth may yield new insights. Extending such experiments to the swarming state of HS2B may shed light on the molecular mechanism of its hyperswarming activities, which will expand the scope of this report.

## MATERIALS AND METHODS

### Bacterial strains.

The Enterobacter sp. SM1_HS2B examined in this study is a swarming-enhanced derivative of the novel bacterial strain SM1 isolated from normal C57BL/6 mouse feces (16). HS2B was derived through a “swarming relay assay” of SM1 on a soft agar plate. Specifically, 2 μL overnight SM1 culture was inoculated onto the center of a 0.5% agar plate (5 g/L NaCl, 5 g/L agar, 10 g/L tryptone, 5 g/L yeast extract), and the plate was incubated at 37°C to facilitate rapid expansion of the colony, i.e., swarming.

Before the swarm colony expanded to reach the periphery of the petri dish, 2 μL of cells from the swarm colony edge was aspirated by a micropipette and inoculated onto the center of a fresh agar plate. After 5 repeats of inoculation and incubation, 2 μL of cells taken from the edge was transferred to 5 mL LB for overnight culture growth. The overnight culture was mixed with glycerol so that the mixture contained 30% glycerol by volume. It was then aliquoted into cryotubes and stored at −80°C for future use. In addition, a wild-type E. coli strain (HCB33) stored in the same fashion was used for comparison in growth of cell length.

### Experimental procedure.

A frozen stock of SM1_HS2B, its control strain SM1, or E. coli strain HCB33 was retrieved briefly from a −80°C freezer and scrubbed for inoculation in a flask containing 10 mL Luria-Bertani (LB) broth (5 g/L NaCl, 10 g/L tryptone, 5 g/L yeast extract). The flask was incubated at 37°C overnight. After 16 h of overnight growth, an aliquot of the bacterial culture was taken and diluted 100-fold into a flask with fresh LB broth and placed in the incubator. After 1 h of incubation at 37°C while being agitated on a shaker at 160 rpm, an aliquot of the fresh culture was diluted again 100-fold into another flask with fresh broth and incubated under the same setting. This broth is named the first regrowth. Each hour later, we took 5 μL of bacteria from the first regrowth for microscopic observation. To do that, the sample was deposited on a microscope slide and then sealed using silicon grease with a cover slit. The hourly observation spanned 6 h as indicated by a flow line next to the left column of images in Fig. 1.

Two hours following the start of the first regrowth, an aliquot of 100 μL was taken and diluted into another fresh flask of 10 mL LB, starting a second regrowth under the same condition of incubation. In each subsequent hour, we took 5 μL of bacteria from this second regrowth, placed it on a microscope slide, sealed it, and observed it as described above. The procedure is indicated by a second flow line, which is next to the representative images in the right column of Fig. 1. Prior to starting each new growth by dilution, a flask with fresh LB broth was prewarmed in the incubator for 30 min so that the cell growth was consistently at 37°C.

Note that our protocol of repeated regrowth of HS2B in liquid medium is designed not to cause selection of yet another new mutant. The entire procedure of even multiple cycles of dilution was routinely performed on a single day. Even if some genetic mutations occur, our protocol includes no mechanism of selection for new mutants. The repeated dilution described above is different from the serial plate inoculation method used to produce the HS2B mutant, under which selection is done at each plating step to preferentially pick those that migrate faster toward the colony edge, i.e., the hyperswarmers.

### Measurement of cell length.

At each time point of the cell growth experiment, multiple phase-contrast images were captured from randomly selected regions within a sample slide. The collections of images were processed using ImageJ or a custom MATLAB script, which identifies cells of different lengths and measures the contour length of all cells in the field.

### Measurements of swimming percentage and swimming speed.

Cell motion was imaged on a Nikon Eclipse TE2000 inverted microscope with a 40× PH2 objective. Images were recorded using a Point Grey Grasshopper 3 camera at the rates of 37 frames per second. Images taken 0.135 s apart were compared by ImageJ to distinguish swimming from nonswimming cells. The swimming speed is calculated as the distance traveled by the cell body divided by the time interval of 0.135 s or longer.

### 3-D cell tracking.

Cell motion was imaged on the same Nikon Eclipse TE2000 inverted microscope with the same camera but using a 20× 0.4-numerical-aperture (NA) PH2 objective. An in-house MATLAB code was used for cell identification and tracking. Variations in pixel intensity Δ*I* along the cell were used to estimate z-offset from the focal plane along the cell body, using Δ*z *= 0.296 × Δ*I*, where Δ*z* (in micrometers) is the change in z-coordinate from the focal plane. Details on 3-D shape reconstruction and rigid-body transformation are provided in the supplemental material.

### Simultaneous measurements of swimming speed and body rotation.

Cells were tracked over at least 3 periods of rotation or 5 s to measure the linear swimming speed and angular speed of cell rotation simultaneously. The average linear speed is calculated as the distance traveled by the centroid of the cell body divided by the time interval. The angular speed is determined as 2*π* divided by the time that a cell takes to complete one revolution. It was averaged over multiple consecutive revolutions, recognized by tracking the cell’s image profile over time. The cell length for each swimming cell was also averaged over multiple images, as the contour length measured in 2-D projection varied over time due to cell orientation and rotation along its long axis.
