# Supplementary material for: Enterobacter sp. Strain SM1_HS2B Manifests Transient Elongation and Swimming Motility in Liquid Medium
Source: Microbiol Spectr. 2022 Jun 1;10(3):e02078-21. doi: 10.1128/spectrum.02078-21 (PMC9241836; doi:10.1128/spectrum.02078-21)
Supplement: SUPPLEMENTAL FILE 5 — Supplemental material. Download spectrum.02078-21-s0005.pdf, PDF file, 2.1 MB [file spectrum.02078-21-s0005.pdf]

Supplemental Materials
Enterobacter sp. SM1_HS2B manifests transient elongation and swimming motility in liquid medium

April 15, 2022

Zhiyu Zhang [a,#], Haoming Liu [a,#], Hamid Karani [a], Jon Mallen [a], Weijie Chen [a,b], Arpan De [b], Sridhar Mani [b], Jay X. Tang [a,*]

[a] Brown University, Physics Department, Providence, RI, USA, [b] Albert Einstein College of Medicine, New York City, USA

# Z. Zhang and H. Liu contributed equally to this work. Author order was determined based on chronology in participation.

* Corresponding author. Email to: jay_tang@brown.edu

S1 Change of average length following repeated regrowth

In order to determine how long HS2B cells may reach upon repeated cycles of regrowth, we performed an additional experiment to make a dilution in fresh LB medium every 2 hours following the previous dilution and growth, and then measure the average cell length under the same growth condition 2 hours after each dilution. For consistency with the experimental procedure described in the main text, we started with a fresh growth from an overnight culture for 1 hour and then made the 1st and 2nd regrowth in 1:100 dilution every 2 hours, which exactly followed the set protocol. In order to maintain high enough cell density for microscopic observation following subsequent steps of dilution, however, we made the 3rd-6th regrowth in 1:10 dilution. We also focused this additional experiment on imaging cells 2 hours after each dilution when the average cell length is known to be at the peak value in each cycle of growth. As shown in Fig. S1, the average cell length has reached its peak of over 50 μm, which sustains over 2 more cycles of dilution and growth but then drops steeply. This additional experiment suggests that the delay in cell division upon infusion of fresh medium lasts for several hours, but not indefinitely.

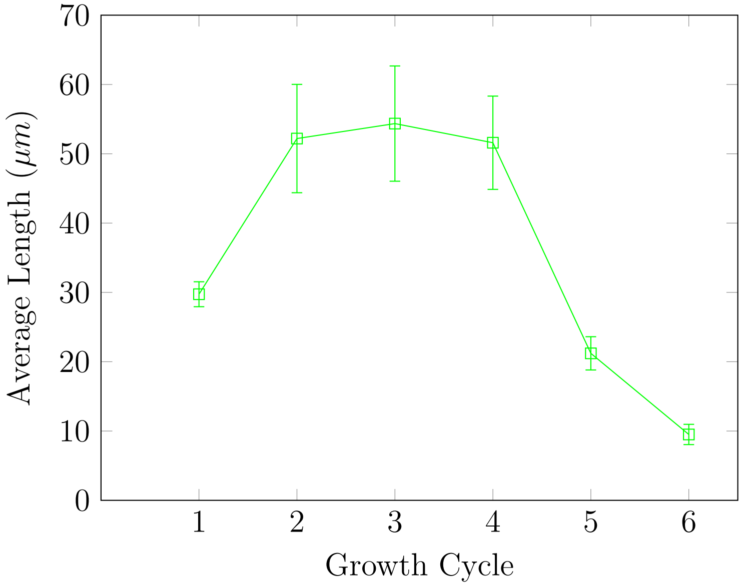

Figure S1: Change of the average cell length of HS2B following several cycles of dilution and growth. The average attains a high value over 50 μm following the 2nd cycle of dilution. It maintains the high value over the cycles of 3 and 4, but then it decreases steeply over additional cycles of dilution and growth. The error bars represent standard deviation of 100 cells measured for each data point.

S2 3D shape reconstruction

The 3D cell shape reconstruction is based on using variation of pixel intensity along the contour to infer the z-offset in cell shape. Towards this end, we extract cell centerline pixel intensities from microscopy images at different focal positions, using a 20x objective lens on a Nikon Eclipse TE2000 inverted microscope but a deliberately mismatched phase 3 ring to yield images of dark-field effects. We use the fine focusing knob on the microscope to change the focal plane at increments of 5 μm, starting from a depth where the images of the cells of interest are over 20 μm out of focus. The difference in centerline pixel intensity ΔI between each focal z-value and its value in the initial image is calculated. The resulting data is plotted against the corresponding z-differences Δz in Fig. S2. The correlation between Δz and ΔI is fitted by a straight line, which results in $\Delta z = 0.296 \times \Delta I$. Although only shown for two cells in Fig. S2, the validity of this linear relation was tested and verified against multiple cases. This linear relation is used to reconstruct cell shape in z-direction from the variation of intensity along the cell centerline.

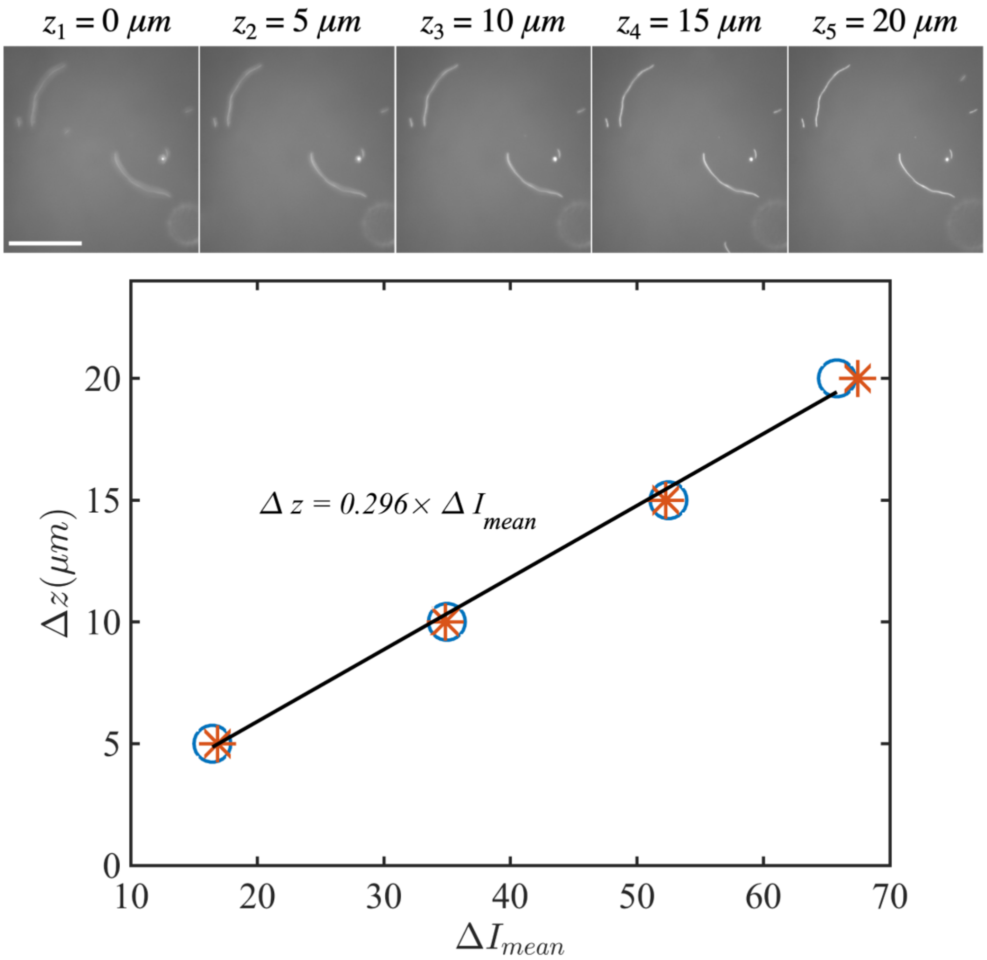

Figure S2: Determination of 3-d contours of elongated bacteria by dark-field microscopy with incremental defocusing. Top row: Phase contrast microscopy images of HS2B cells at different focal positions. Focal plane was changed from $z = 0\ \mu m$ using fine focusing knob at an increment of $5\ \mu m$. The corresponding pixel intensity at cell center-line was extracted using the method outlined under 3D Cell Tracking sub-section in the Materials and Method section of the paper. Scale bar is 100 μm. Bottom row: Calibration plot showing variation in pixel intensity with z-offset of the bacterial images in top row. The $\circ$ and $*$ correspond to pixel intensity data of the two long cells shown in the images above.

S3 Fluorescence imaging and cell labeling

We labeled HS2B cells with the fluorescent protein dye Nano Orange (Thermo Fisher Scientific, Inc.) with the goal of monitoring the onset of cell division as HS2B cells elongate in fresh medium. In order to enhance the binding of Nano Orange with cells, the samples were mixed with non-ionic detergent Triton X-100 [4, 7]. Briefly, 20-50 μL of 1M Triton X-100 was added to 1 mL of live bacterial culture and the solution was mixed by pipetting several times. Then, 0.2 μL of concentrated Nano Orange reagent was added directly to 10 μL of the bacterial solution on a microscope glass slide and then sealed with a cover slip and vacuum grease. The sample was placed in a dark room for about 30 min before imaging. Cells were imaged under a Nikon Eclipse TE2000 inverted microscope with a 60x-1.4NA oil immersion objective and a fluorescence filter cube with blue excitation and green emission wavelengths.

Labeled HS2B cells show regular septa (indicated by arrow heads in Fig. S3) formed along the body of the cell. Occasionally, kinks are noticeable (indicated by empty arrow heads on the inset of the middle panel in Fig. S3), which provide further evidence on the fate of most elongated, filamentous cells. Curiously, we noted that Nano Orange is prone to bind only a subset of cells among those that are over 20 μm. For instance, both the middle and the right panels in Fig. S3 (among numerous images not shown) highlight

the preferential staining of long HS2B cells.

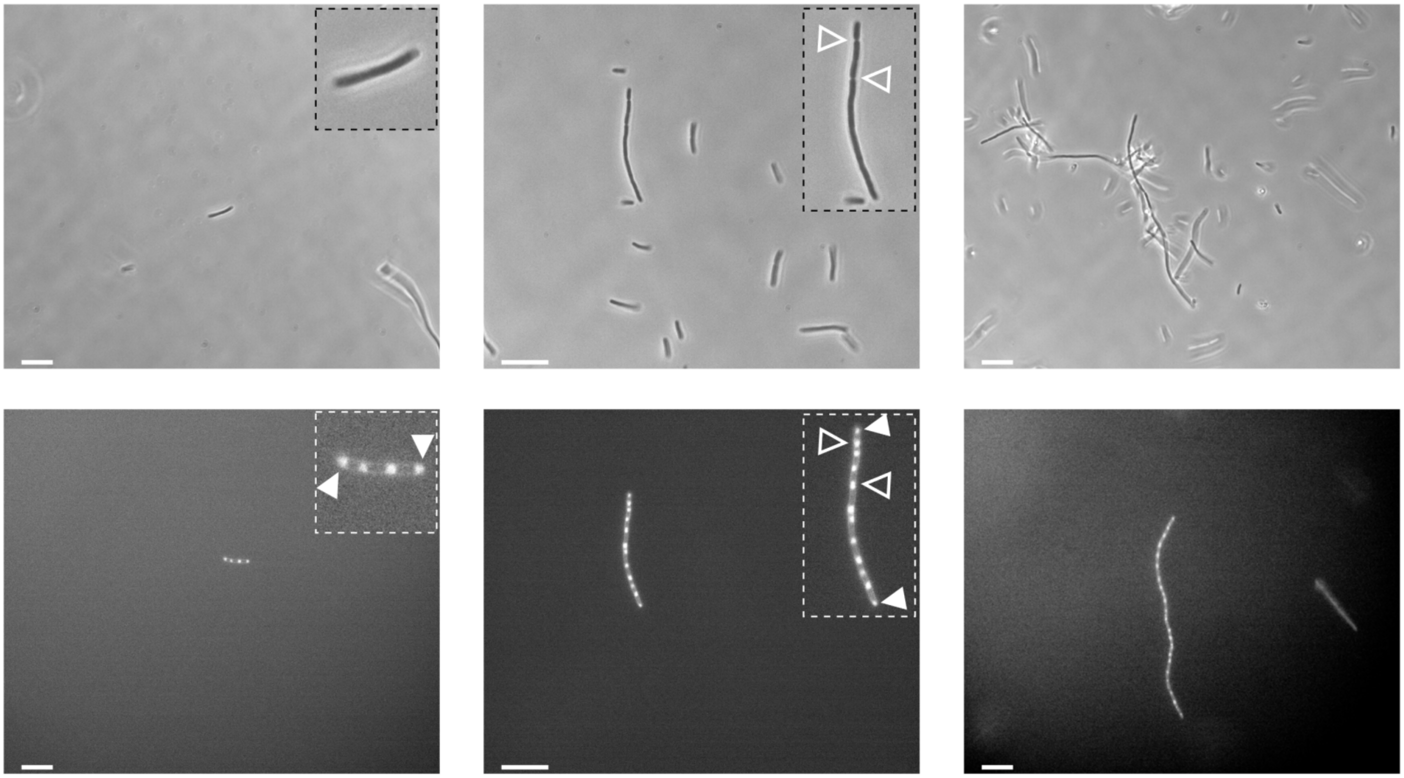

Figure S3: Microscopy evidence of long cells consisting of septa showing a delay in cell division. Top row: Phase contrast microscopy images of HS2B cells. Bottom row: Fluorescence microscopy images of the same cells stained with the Nano Orange protein dye. Insets: Magnified images of selected cells within their respective large field images. Filled triangles mark fluorescence localization at the poles and open triangles indicate fluorescence localization at kinks (constriction sites). Differences in cell orientation between phase contrast and fluorescence images are due to cell movement during the time the counterpart images were acquired. Scale bar indicates 10 μm.

Most cells stained with Nano Orange augmented by Triton X-100 show regular septa. In some fluorescent images, however, we observed a heterogeneous mixture of cells with and without labeled septa, an example of which is shown in Fig. S4.

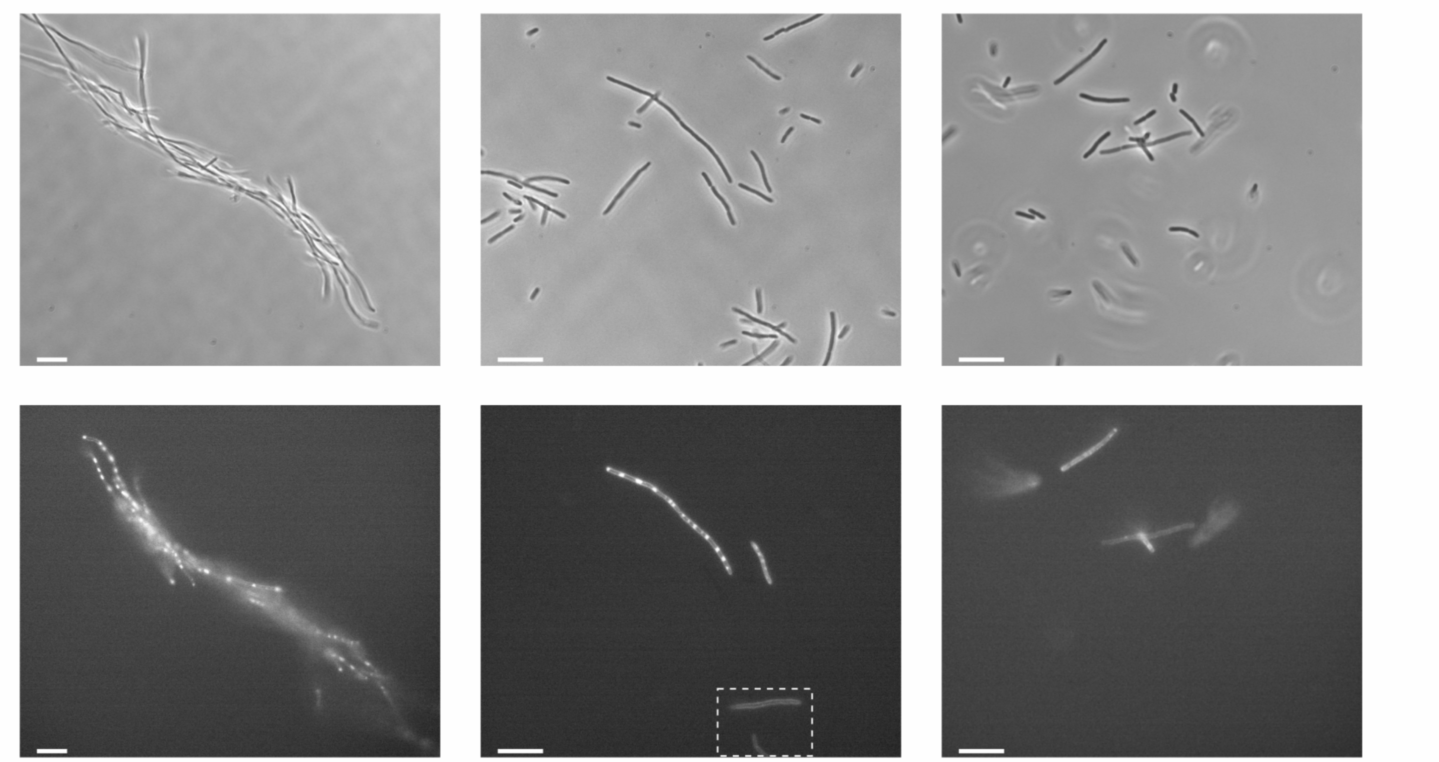

Figure S4: Mixed groups of cells with varied levels of fluorescence labelling. Top row: Phase contrast microscopy images of HS2B cells. Bottom row: Fluorescence microscopy images of the same cells stained with the NanoOrange protein dye. While most labeled cells show regular septa, some cells (for instance, middle panel in a rectangular box and those in the right panel) are only labeled on the surface. The notable difference in orientation between the labelled cell marked inside the rectangular box and its corresponding phase contrast image is due to cell movement during the time the counterpart images were acquired. Scale bar is 10 μm.

S4 Imaging flagella by negative staining electron microscopy

To visualize flagella that propel HS2B cells in liquid medium, we prepared bacterial samples for negative staining electronic microscopy. Overnight HS2B bacteria were regrown for 2.5 h and 1:1 mixed with the fixatives made up of 5% glutaraldehyde, 4.0% paraformaldehyde, in 0.2 M sodium Cacodylate buffer. Carbon-only grids of 400 mesh size were plasma cleaned using a Tergeo-EM Plasma Cleaner (PIE Scientific, USA). The bacteria contained in a droplet applied to the grid were then negatively stained with 1% Uranyl Acetate and viewed on a Tecnai 20 transmission electron microscope (Thermo Fisher) at 120 kV. The result is shown in Fig. S5.

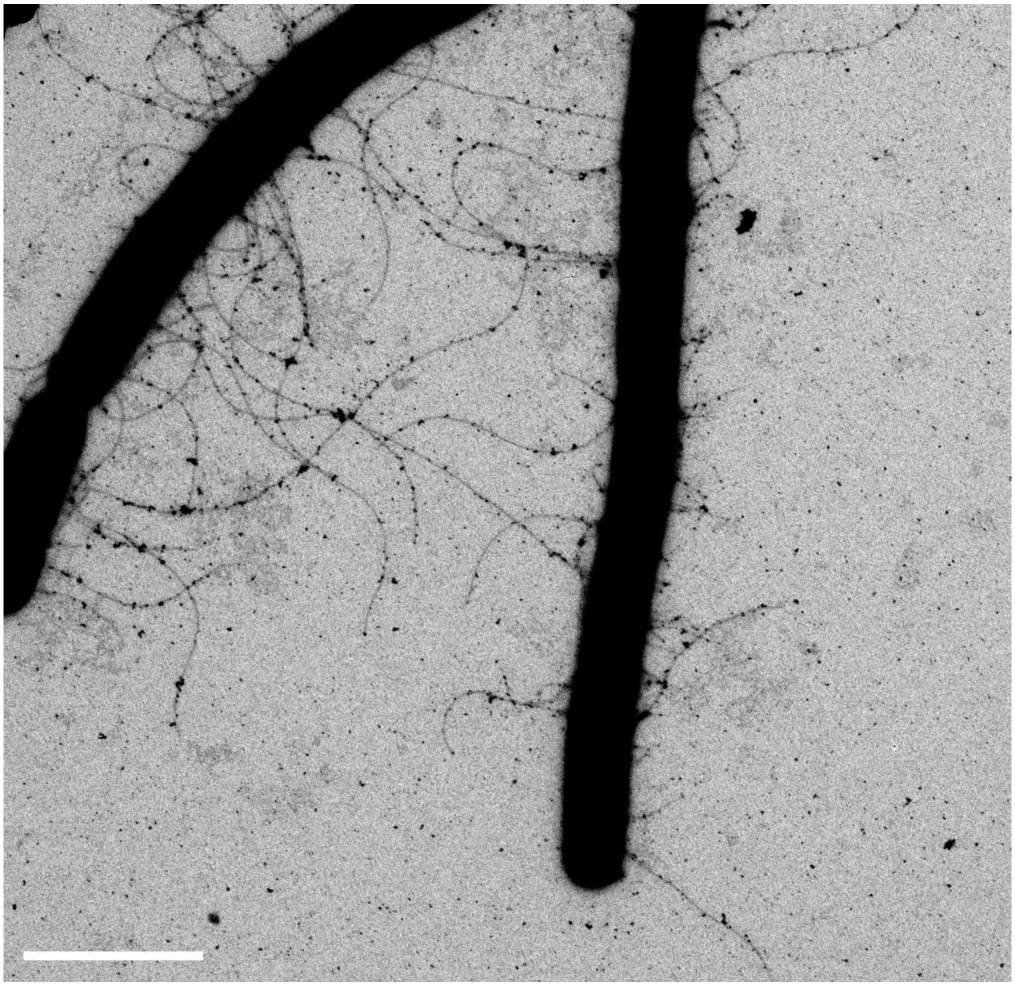

Figure S5: An electron microscopy image showing negatively stained flagella filaments of elongated SM1_HS2B cells. Cells grown in 37 °C were harvested from a 2.5 h regrowth of the overnight culture. Scale bar on the lower left is 2 μm.

S5 Hydrodynamic modeling and torque calculations

To calculate the torques exerted on a cell swimming in liquid medium, we approximated it as either a slender rod-like body (Fig. S6 green carets) or a prolate spheroid (Fig. S6 red and blue carets) rotating along the major axis. Denote $\mathrm{L_a}$ half of the length L of the cell. The semi-minor axis $\mathrm{L_b}$ of the spheroidal cell body is set to be 1μm (half of a typical cell's width). The errors resulted from both models are of the order $O((\mathrm{L_b}/\mathrm{L_a})^3)$.

As the first order approximation, we used an analytical, closed-form formula for a slender rod rotating in static fluid [2] to obtain the cell torque as

$$\mathrm{T_{uni}} = 8\pi\mu \mathrm{L_a}{\mathrm{L_b}}^2(1 - \frac{\mathrm{L_b}}{2\mathrm{L_a}} + \frac{{\mathrm{L_b}}^2}{2{\mathrm{L_a}}^2})\Omega,$$

where μ is the viscosity of water and Ω is the angular speed of body rotation with respect to its long axis.

The expression above is then compared with a prolate spheroid model. For a prolate spheroid rotating in static fluid [2], the torque is

$$\mathrm{T_{non-uni}} = 32\pi\mu \mathrm{L_a}\epsilon\beta_0\Omega,$$

where $\epsilon = \sqrt{1 - \frac{b^2}{a^2}}$ is the eccentricity and $\beta_0 = {\mathrm{L_a}}^2\epsilon^2(\frac{2\epsilon}{1-\epsilon^2} - \log\frac{1+\epsilon}{1-\epsilon})^{-1}$.

Lastly, for a prolate spheroid translating while rotating in liquid medium, the closed-form formula for the torque exerted on the body is given by Happel & Brenner as [5]

$$\mathrm{T_{HB}} = 8\pi\mu{\mathrm{L_b}}^3(\frac{2}{5} + \frac{\mathrm{L_a}}{\mathrm{L_b}})\Omega.$$

Note that, just like the two calculations above it for a body rotating but not translating in liquid medium, the torque $\mathrm{T_{HB}}$ for a prolate spheroid both rotating and translating remains proportional to the rotation speed Ω.

Utilizing the measured linear and angular speeds of HS2B, we calculate the torque experienced by the cell body for 359 cells. By modelling cells each as a slender body rotating and translating through low Reynolds number fluid, we use the analytic equations above for both slender rod and ellipsoid to find approximate values for torque [2, 5]. We then compare the torque calculated for HS2B cells with torque produced by different bacterial species, such as *E. coli* and Spirochetes. The reported flagellar motor torque for *E. coli* varies from a low end value of 370 pNnm [3] to a high end value of 4600 pNnm measured with optical tweezers [1]. This high value, however, is deemed unreliable later and a lower value of 1260 pNnm was reported [9]. This corrected value has since been suggested as the motor torque for *E. coli* [10]. The reported values for spirochetes are species dependent, which are 800 pNnm for Treponema pallidum [6], 2700 pNnm for Borrelia burgdorferi [6], and 4000 pNnm for Leptospira [8]. These values, as well as those for three strains of *E. coli*, are indicated in Fig. S6 for close comparison with this study on HS2B. For the three different analytic equations corresponding to the quoted models, the values calculated for the torque generated by HS2B cells are comparable (Fig. S6 colored carets). At the same length scale of ($\sim$10 μm), HS2B produces larger torque than that produced by most species of spirochetes (Fig. S6 grey symbols) [6, 8], although the range of values for the latter is rather wide, casting some doubt on their reliability. The medium value out of the 3 indicated for *E. coli*, as argued above to be the value of choice for comparison, appears consistent with the down trend of the HS2B data as the cell length decreases. This comparison is less direct since all HS2B cells measured in this report are much longer than *E. coli*.

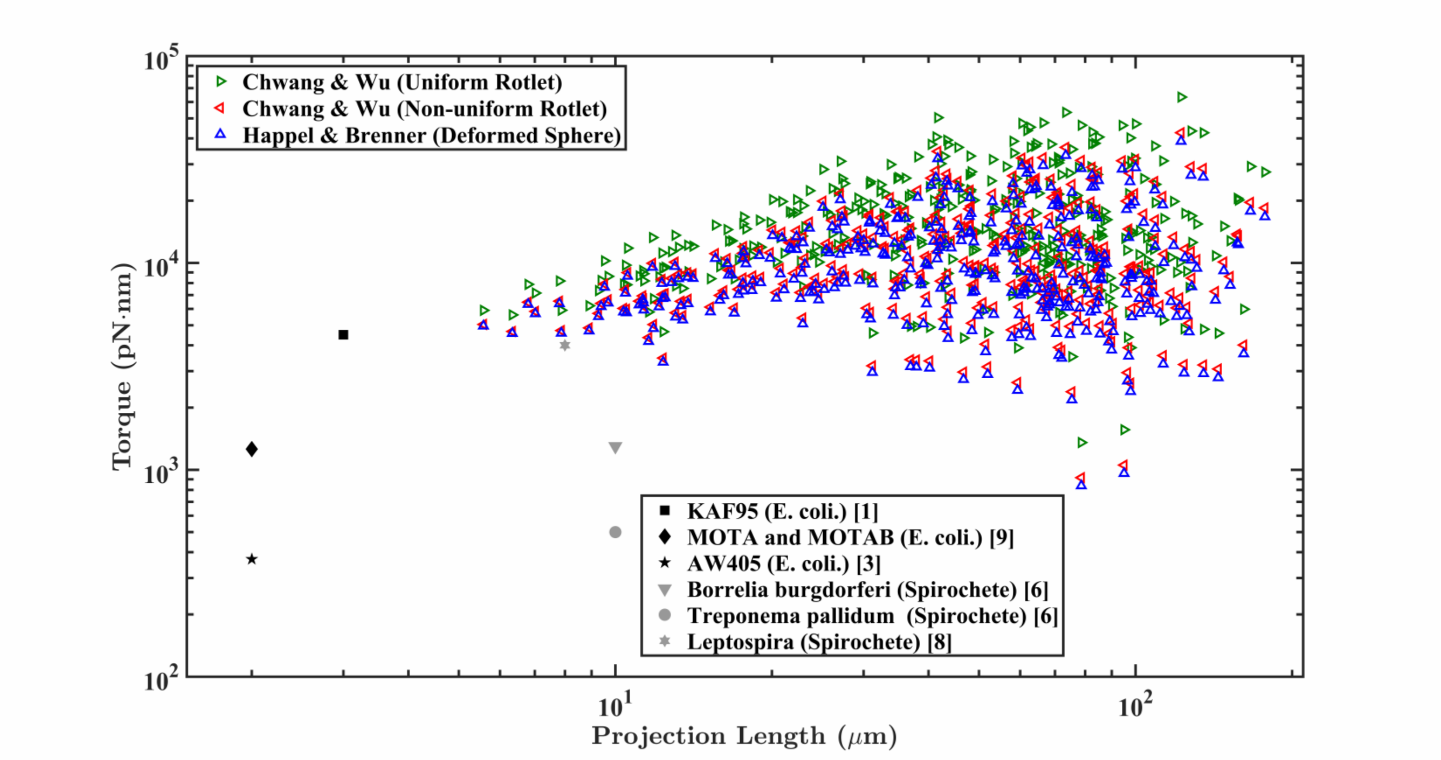

Figure S6: Torque of HS2B of various length compared with those reported for *E. coli* and Spirochete. A total number of 359 HS2B cells were measured with their rotation rate and swimming speed shown in Fig. 6 of the main paper. The torque is calculated for each cell based on its measured length and rotation speed using the hydrodynamic models of Chwang & Wu [2] and Happel & Brenner [5].

S6 Supplemental Movies

Movie S1: Elongated HS2B cells of varied length move rigorously and, with rare exceptions, unidirectionally. Video plays in real time.

Movie S2: A sudden change in motion of a long HS2B cell, from self-rotating but not translocating to translocating with nearly constant speed. Video plays in real time.

Movie S3: A swimming, elongated cell divided into two swimming cells. Video plays in real time.

Movie S4: HS2B cells swim by rigid-body rotation, confirmed by overlaying its 3D contour superimposed on the rotating and translating elongated cell (shown in Fig. 5 of the main paper). They do not swim by lateral undulation, in spite of the visual appearance consistent with either possibility. Video plays in real time.

References

[1] R. M. Berry and H. C. Berg. Absence of a barrier to backwards rotation of the bacterial flagellar motor demonstrated with optical tweezers. *Proceedings of the National Academy of Sciences*, 94(26): 14433–14437, 1997. doi: 10.1073/pnas.94.26.14433. URL `https://www.pnas.org/content/pnas/94/26/14433.full.pdf`.

[2] A. T. Chwang and T. Y.-T. Wu. Hydromechanics of low-reynolds-number flow. part 1. rotation of axisymmetric prolate bodies. *Journal of Fluid Mechanics*, 63(3):607–622, 1974. ISSN 0022-1120. doi: 10.1017/S0022112074001819. URL `https://www.cambridge.org/core/article/hydromechanics-of-lowreynoldsnumber-flow-part-1-rotation-of-axisymmetric-prolate-bodies/4FF9DD56BD26D48C79FDD07D526A7899`.

[3] N. C. Darnton, L. Turner, S. Rojevsky, and H. C. Berg. On torque and tumbling in swimming escherichia coli. *Journal of Bacteriology*, 189(5):1756–1764, 2007. ISSN 0021-9193. doi: 10.1128/JB.01501-06. URL `https://jb.asm.org/content/189/5/1756`.

[4] H. P. Grossart, G. F. Steward, J. Martinez, and F. Azam. A simple, rapid method for demonstrating bacterial flagella. *Appl Environ Microbiol*, 66(8):3632–6, 2000. ISSN 0099-2240 (Print) 0099-2240. doi: 10.1128/aem.66.8.3632-3636.2000.

[5] J. Happel and H. Brenner. *Low Reynolds number hydrodynamics: with special applications to particulate media*, volume 1. Springer Science & Business Media, 1983. ISBN 9024728770.

[6] M. Harman, D. K. Vig, J. D. Radolf, and C. W. Wolgemuth. Viscous dynamics of lyme disease and syphilis spirochetes reveal flagellar torque and drag. *Biophysical journal*, 105(10):2273–2280, 2013. ISSN 1542-0086 0006-3495. doi: 10.1016/j.bpj.2013.10.004. URL `https://pubmed.ncbi.nlm.nih.gov/24268139https://www.ncbi.nlm.nih.gov/pmc/articles/PMC3838743/`.

[7] L. J. Jones, R. P. Haugland, and V. L. Singer. Development and characterization of the nanoorange protein quantitation assay: a fluorescence-based assay of proteins in solution. *Biotechniques*, 34(4): 850–4, 856, 858 passim, 2003. ISSN 0736-6205 (Print) 0736-6205. doi: 10.2144/03344pt03.

[8] S. Nakamura, A. Leshansky, Y. Magariyama, K. Namba, and S. Kudo. Direct measurement of helical cell motion of the spirochete leptospira. *Biophysical journal*, 106(1):47–54, 2014. ISSN 1542-0086 0006-3495. doi: 10.1016/j.bpj.2013.11.1118. URL `https://pubmed.ncbi.nlm.nih.gov/24411236https://www.ncbi.nlm.nih.gov/pmc/articles/PMC3907252/`.

[9] S. W. Reid, M. C. Leake, J. H. Chandler, C.-J. Lo, J. P. Armitage, and R. M. Berry. The maximum number of torque-generating units in the flagellar motor of *Escherichia coli is* at least 11. *Proceedings of the National Academy of Sciences*, 103(21):8066–8071, 2006. doi: 10.1073/pnas.0509932103. URL `https://www.pnas.org/content/pnas/103/21/8066.full.pdf`.

[10] Y. Sowa and R. M. Berry. Bacterial flagellar motor. *Quarterly reviews of biophysics*, 41(2):103–132, 2008.
